# Bullying victimization among Lebanese adolescents: The role of child abuse, Internet addiction, social phobia and depression and validation of the Illinois Bully Scale

**DOI:** 10.1186/s12887-020-02413-1

**Published:** 2020-11-14

**Authors:** Diana Malaeb, Emmanuelle Awad, Chadia Haddad, Pascale Salameh, Hala Sacre, Marwan Akel, Michel Soufia, Rabih Hallit, Sahar Obeid, Souheil Hallit

**Affiliations:** 1 School of Pharmacy, Lebanese International University, Beirut, Lebanon; 2 Faculty of Psychology, University of Lund, Lund, Sweden; 3 Research and Psychology Departments, Psychiatric Hospital of the Cross, Jal Eddib, Lebanon; 4 Neuroépidémiologie Tropicale, Institut d’Epidémiologie et de Neurologie Tropicale, Université de Limoges, UMR 1094, GEIST, 87000 Limoges, France; 5 INSPECT-LB: Institut National de Santé Publique, Épidémiologie Clinique et Toxicologie-Liban, Beirut, Lebanon; 6 Faculty of Pharmacy, Lebanese University, Hadat, Lebanon; 7 Faculty of Medicine, University of Nicosia, Nicosia, Cyprus; 8 Faculty of Medicine and Medical Sciences, Holy Spirit University of Kaslik (USEK), Jounieh, Lebanon; 9 Faculty of Medicine , Lebanese University , Hadat, Lebanon; 10 Department of Infectious Disease , Bellevue Medical Center , Mansourieh, Lebanon; 11 Department of Infectious Disease, Notre Dame des Secours University Hospital , Byblos, Lebanon; 12 Faculty of Arts and Sciences , Holy Spirit University of Kaslik (USEK) , Jounieh, Lebanon

**Keywords:** bullying victimization, child abuse, Internet addiction, social anxiety, adolescents

## Abstract

**Background:**

Both bullying victimization and perpetration were associated with depression, social phobia, physical and psychological child abuse and Internet addiction in Lebanon. The prevalence of bullying in Lebanon is alarming, with 50% of school-aged children and adolescents reporting being bullied at some point. The high rate of both bullying victimization can be reflective of the inefficacy of current prevention and intervention policies in targeting associated problematic individual and contextual factors. The objective of the present study was to analyze factors associated with bullying victimization and validate the Illinois Bully Scale among Lebanese adolescents.

**Methods:**

This is cross-sectional study that took place between January and May 2019. We enrolled 1810 adolescents between 14 and 17 years of age. The Illinois Bully scale was used to measure bullying victimization. In order to ensure the adequacy of the sample with values greater than 0.8 - an indicator that component or factor analysis was useful for these variables - we used Kaiser-Meyer-Olkin (KMO) measurement. Statistical significance considered if the p-value < 0.05.

**Results:**

The results showed that 841 (46.5%, CI: 44.1% – 48.7%) participants were classified as having been previously bullied. None of the bullying scale items was removed. Items on the bullying scale converged on a two-factor solution with Eigenvalues greater than 1, accounting for a total of 73.63% of the variance (Factor 1: bullying victimization; Factor 2: bullying perpetration; KMO = 0.899, Bartlett’s sphericity test p < 0.001; αCronbach = 0.955). Having a separate parents (ORa = 3.08), Mild (ORa-4.71) to moderate (ORa = 3.84) internet addiction test, higher social fear (ORa = 1.50), higher psychological abuse (ORa = 3.59), higher child neglect (ORa = 2.21) and physical (ORa = 4.55) abuse were significantly associated with higher odds of being bullied. However, higher social avoidance (ORa = 0.49), poor (ORa = 0.20), fair (ORa = 0.94) and very good (ORa = 0.04) physical activity as compared to sedentary were significantly associated with lower odds of being bullied.

**Conclusions:**

Our findings attest that bullying victimization is likely to be associated with certain factors such as child abuse of all forms, Internet addiction, social fear and avoidance. In addition, the Illinois Bully Scale was validated in Lebanon. More attention should be paid to students vulnerable to bullying victimization, such as those with environmental or domestic problems, and adolescents with psychological disorders such as behavioral addictions.

## Background

Bullying is defined as a harmful, aggressive, intentional, and repeated negative behavior by peers directed against a person who has difficulty defending oneself [1]. It is one of the most common phenomena involving a power imbalance, across countries [2, 3]. Recently, bullying among children and adolescents has been recognized as an increasingly growing problem, constantly emerging in new forms [4]. Bullying can be perpetrated directly or indirectly [5, 6]. Direct forms of bullying include threatening, embarrassing, physically harming and verbally pressuring others [5, 7]. On the other hand, indirect forms of bullying can involve anonymously destroying another’s social reputation, social alienation and spreading rumors about others [8, 9]. Although bullying can occur among all age groups, the majority of research conducted focused on adolescents in schools [8]: 10–30% of adolescents in Australia, Europe, United States, and Latin America are involved in bullying behaviors [10–15]. It has been reported in several surveys that 8–70% of children and adolescents were victims of bullying [16, 17]. The prevalence of bullying victimization varies among studies of different populations, which could be explained by diverse factors, such as the difference in definitions of bullying and methodology used in each study [18]. The consequences of bullying can be highly devastating; it can cause severe health problems, antisocial behavior, adaptation problems and psychiatric disorders [19–21].

Factors that increase the risk of bullying victimization encompass both the adolescent and his/her environment [18]. The extent of bullying exposure appears to be equal in both sexes, however, males are more exposed to physical bullying while females engage in spreading rumors and ostracism [22]. There are many factors associated with bullying victimization such as depression, Internet addiction, physical, sexual, and psychological abuse, psychoactive substance use, domestic violence, low socioeconomic status, a high pressure environment and unstable relations with peers [23–26].

### Bullying victimization and depression

There is substantial evidence associating bullying with depression; bullying victimization positively correlated with depressive symptoms in adolescents [27]. Also, depression was a baseline predictor and an outcome of bullying victimization [28].

### Bullying victimization and Internet addiction

Internet addiction was linked to being a victim of bullying, specifically cyberbullying [29], while also predicting traditional in-person bullying victimization [30]. Inversely, bullying victimization predicted Internet addiction [31].

### Bullying victimization and child abuse

It is important to mention the relationship between child abuse and bullying victimization: adolescents who were sexually abused had a higher probability of being victims of bullying [32]. Similarly, physical child abuse increased the odds of bullying victimization [33]. Overall, child psychological, physical and sexual abuse and neglect were related to both bullying victimization and perpetration [34].

### Bullying victimization and social phobia

Children who report social phobia display persistent and extreme phobia of social situations, mainly caused by a negative evaluation from others, which generates intense fear [13]. Individuals with social phobia also report fear and avoidance caused by scrutiny, judgment or humiliation inflicted by others. Furthermore, adolescents who are victims of bullying were more likely to suffer from psychological disorders, notably social phobia [35]. A study found a bidirectional relationship exists between social phobia and bullying victimization [36].

**Fig. 1:**
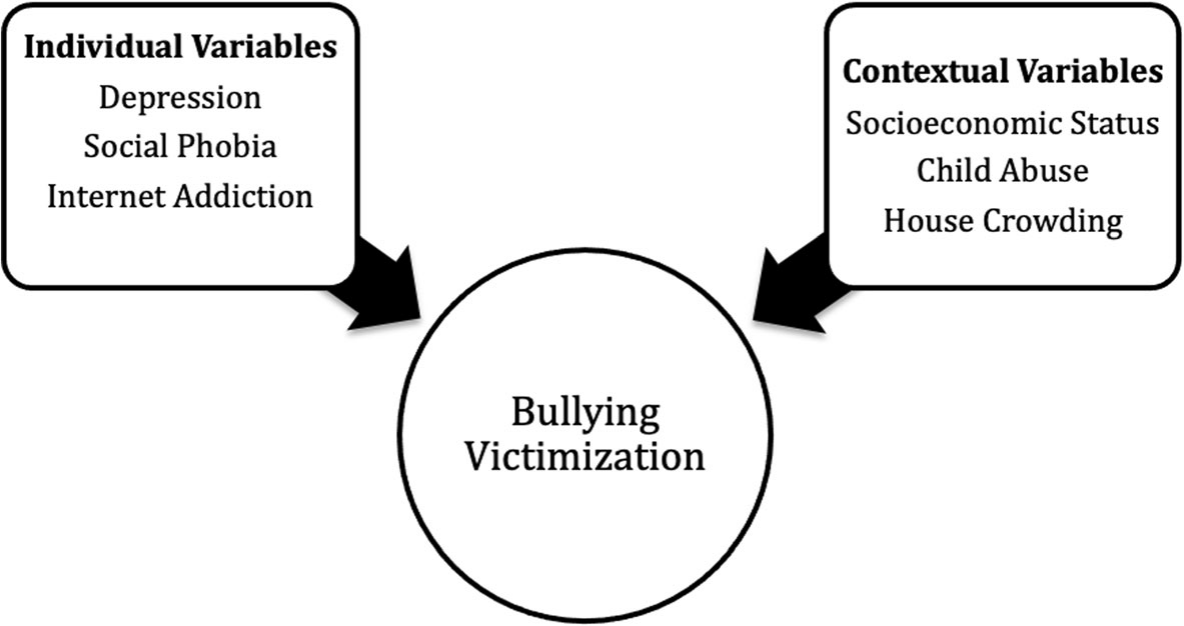
Individual and contextual variables associated with bullying victimization

Since bullying is associated with many potential factors, a conceptual framework was previously developed to investigate the association between bullying and substance misuse [37]. The figure below shows the factors that affect the risk of bullying victimization, based on the previous proposed conceptual framework pertaining to bullying [37] (Fig. 1).

### Bullying victimization assessment measures

Many bullying assessment measures exist, with some measuring bullying victimization, bullying perpetration or both bullying victimization and perpetration [38]: the California Bullying Victimization Scale exclusively measuring victimization [39], some studies have compiled bullying perpetration items from different scales [40] while the Olweus Bully/Victim Questionnaire measures both aspects of bullying [41]. Bullying assessment measures for adolescents and school students are in high demand, with call for evidence-based scales becoming increasingly needed [42]. The Illinois Bully Scale has demonstrated good validity and reliability in China [43], Italy [44], Estonia [45], Iran [46] and Pakistan [47]. Due to its exemplary psychometric quality in Asian and Middle Eastern populations, it was deemed valuable to validate the Illinois Bully Scale in Lebanon, a Middle Eastern country, in its native language Arabic.

### Purpose of the study

Recently, both bullying victimization and perpetration were associated with depression, social phobia, physical and psychological child abuse and Internet addiction in Lebanon [48]. It is estimated that 13.1% of Lebanese adolescents suffer from mood disorders, including depression [49]. In Lebanon, bullying victimization had a significant relationship with anxiety disorders [50]. According to previous studies conducted on Lebanese school aged children, bullying is associated with and socioeconomic and social factors as young age, low family income, low parental education, private tutoring, higher alcohol consumption and smoking [50, 51]. The prevalence of bullying in Lebanon is alarming, with 50% of school-aged children and adolescents reporting being bullied at some point [52]. The high rate of both bullying victimization can be reflective of the efficacy of current prevention and intervention policies in targeting associated problematic individual and contextual factors. Therefore, the objective of the present study was to analyze factors associated with bullying victimization and validate the Illinois Bully Scale among Lebanese adolescents. We hypothesize that similar to other populations around the world, bullying victimization is associated with negative individual and contextual variables in Lebanon.

## Methods

### Study design

This research was conducted using a cross-sectional design from January until May 2019. A simple randomization method was followed to choose schools from an exhaustive list obtained from the Ministry of Education and Higher Education. Eighteen schools were chosen proportionately according to the districts (Beirut, Mount Lebanon, Central, South and Bekaa). All eligible students from each school were asked to enroll in this study. The methodology used is the same one used in previous papers [53–56].

### Questionnaire

The Arabic questionnaire required on average 60 minutes for completion. The questionnaire was filled by participants at school to avoid any parental involvement while replying. The first part collected sociodemographic information including age, gender, smoking status and parents’ socioeconomic status. The household crowding index was calculated by dividing the number of people living in the same house with the number of rooms in the house, excluding the bathroom and kitchen [57]. The higher the house crowding index, the lower the socioeconomic status of the family. The physical activity index was calculated by multiplying the frequency of exercise by the intensity and the duration [58]. The physical activity index is categorized into five groups: under 20 sedentary, between 20 and 39 poor, between 40 and 59 fair, between 60 and 80 very good and over 80 high.

The second part included:

### The Illinois Bully scale (IBS)

Permission to use the scale was obtained from Dr Dorothy Espelage. It is a research-validated tool used to measure bullying victimization through direct survey [59]; consists of eighteen item scale with two subscales including bullying perpetration (I annoyed other students) and bullying victimization (Other students beat and pushed me). Questions were scored as follows: never = 0 and up to seven times or more = 4. Subscale scores are computed by summing the respective items. Higher bullying perpetration and victimization scores indicated higher bullying perpetration and victimization respectively [60]. In this study, we used only the victimization bullying subscale of the IBS.

### Liebowitz Social Anxiety Scale (LSAS)

This tool includes 24 items, reported on a Likert scale from 0 to 3. The items are divided into two subcategories: 13 questions examining performance anxiety and 11 concerning social situations [61, 62]. Higher scores indicating higher fear and avoidance.

### Internet Addiction Test (IAT)

This test is a 20-item scale that measures the presence and severity of Internet dependency. Each statement is scored as follows: 0 = less extreme behavior to 5 = most extreme behavior. Higher scores reflect higher internet addiction.

### The adolescent depression rating scale (ADRS)

This 10-item scale is used to screen for depression among adolescents, using yes or no questions. The higher the scores, the higher the level of depression will be [63].

### Child abuse self-report scale (CASRS)

This scale displays 38 items divided into four categories of child abuse: psychological (14 items), neglect (11 items), physical (8 items) and sexual (5 items). The responses are reported as follows: 0 = Never 3 = Always [64]. Higher scores indicate more abuse in all subscales [65].

### Translation procedure of the questionnaire

The translation for all scales was achieved using the forward and backward method. A translator conducted the translation from English to Arabic, while another conducted the process from Arabic to English. Translation inconsistencies were resolved by consensus.

### Minimal sample size

According to the G-power software, a prior analysis was done to calculate the sample size and based on an effect size f2 = 2%, a 5% alpha error, and 80% power, and taking into consideration 11 factors to be entered in the multivariable analysis, the results showed that a minimal number of 395 was needed. Also, in the logistic regression, the smallest proportions of those who were not being bullied was 46.7% and the number of covariates used was 11 then the minimum number of participants to include was: *N* = 10*11/0.46 = 239 [66]. Out of 2000 questionnaires distributed; 1810 (90.5%) were completed and collected back.

### Statistical analysis

The 25th version of SPSS software was employed to conduct the data analysis. In the absence of cut off points for bullying victimization scale, LSAS scale, CASRS scale the median was considered as cut off points for low and high score. As the victimization bullying scale was moderately skewed (Skewness value = 0.508) we transformed the variable to log (variable) to get a variable with a more symmetric distribution [67]. The victimization bullying scale was not normally distributed as checked by the Shapiro Wilk test. The non-parametric tests were used. The Spearman correlation analyses were used for continuous variables. We used the Mann-Whitney test to compare dichotomous variables and the Chi-square/Fisher exact tests for categorical variables. A stepwise linear regression was conducted, taking the bullying victimization total score as the dependent variable. Also, a logistic regression analysis taking being bullied vs. not being bullied as the dependent variable was conducted. Bonferroni corrected *p*-value was used to compensate for multiple testing by dividing the p-value by the number of factors to be tested 0.05/13 = 0.004 [68]. The level of significance for the multivariable model was set to *p* < 0.05.

The exploratory factor analysis (EFA) was conducted on half of the sample (*N* = 905) to explore the construct validity of the scale using the principal component analysis technique. The EFA was used to determine the number of factors that underlie the set of items and afterward the confirmatory factor analysis was used to evaluate whether measures of the construct are consistent with the results from the EFA construct (or factor) [67]. The Kaiser-Meyer-Olkin (KMO) measurement of sampling adequacy and Bartlett’s sphericity test were appropriate. The factors retained corresponded to Eigenvalues greater than one. The promax rotation was used as the items were correlated.

Second, a confirmatory factor analysis was carried out using the STATISTICA software on the second half of the sample (Sample 2; *N* = 905). We also reported several goodness-of-fit indicators: the Relative Chi-square (χ2/df), the Root Mean Square Error of Approximation (RMSEA), the Goodness of Fit Index (GFI) and the Adjusted Goodness of Fit Index (AGFI). The value of χ2 divided by the degrees of freedom (χ2/df) has a low sensitivity to sample size and may be used as an index of goodness of fit (cut-off values:<2–5). The RMSEA tests the fit of the model to the covariance matrix. As a guideline, values of < 0.05 indicate a close fit and values below 0.11 indicate an acceptable fit. The GFI and AGFI are Chi-square-based calculations independent of degrees of freedom. The recommended thresholds for acceptable values are ≥ 0.90 [69]. A Cronbach’s alpha was recorded for the scales’ reliability analysis. The whole sample (*n* = 1810) was used to evaluate correlates of bullying victimization scale.

## Results

The Cronbach’s alpha values of the used scales was as follows: for the bullying perpetration scale was 0.971 for the bullying victimization scale was 0.955, for the LSAS scale was 0.975, for the fear subscale was 0.952, for the avoidance subscale was 0.951, for the IAT was 0.925, for the ADRS was 0.940 and for each subscale of the CASRS was psychological (0.973), neglect (0.971), physical (0.966) and sexual (0.954). Table 1 summarizes the sociodemographic characteristics of the participants. The mean age was 15.42 ± 1.14 years, with 53.3% females and 25.9% smokers. In addition, 11.9% of the adolescents had separated/divorced parents. The log mean bullying victimization score in our sample was 1.34 ± 1.28 (median = 1.38). The median was used as the cutoff point in the absence of a cutoff score for this scale; the results showed that 841 (46.5%, CI: 44.1% – 48.7%) participants were classified as having been previously bullied.

**Table 1:** Sociodemographic characteristics of the sample population (*N* = 1810)

	Frequency (%)
**Gender**	
Male	844 (46.7%)
Female	963 (53.3%)
**Parents status**	
Living together	1581 (88.1%)
Separate	213 (11.9%)
**Smoking status**	
Yes	468 (25.9%)
No	1342 (74.1%)
	**Mean ± SD**
**Age (years)**	15.42 ± 1.14
**Household crowding index**	1.01 ± 0.64

### Validation of the bullying victimization score

#### Factor analysis

None of the bullying scale items was removed. We ran the factor analysis on half of the original sample (*n* = 905). Items on the bullying scale converged on a two-factor solution with Eigenvalues greater than 1, accounting for a total of 73.63% of the variance (Factor 1: bullying victimization; Factor 2: bullying perpetration; KMO = 0.899, Bartlett’s sphericity test *p* < 0.001; αCronbach = 0.955) (Table 2).

**Table 2:** Principal component analysis results of the promax rotation of the bullying victimization scale

Question	Item	Factor 1	Factor 2
I was threatened by other students.	14	0.987	
I got hit and pushed by other students.	13	0.977	
Other students called me names.	12	0.943	
Other students called me “gay.”	11	0.859	
Students spread rumors or told lies about me.	15	0.851	
I was excluded or kept out of a group of friends on purpose.	16	0.849	
Other students picked on me.	10	0.827	
I spread rumors about other students.	3		1.021
In a group, I teased other students.	2		0.847
I upset other students for the fun of it.	1		0.844
I started (instigated) arguments or conflicts.	4		0.844
I was mean to someone when I was angry.	9		0.823
I helped harass other students.	5		0.672
I teased other students.	8		0.629
I encouraged people to fight.	7		0.577
I threatened to hurt or hit another student.	6		0.556
**Percentage of variances explained**		73.23	6.99
**Cronbach alpha**		0.971	0.955

#### Confirmatory analysis

The confirmatory analysis was run over the second half of the sample (n = 905) using the one-factor solution obtained from sample 1. The following results were obtained: the Maximum Likelihood Chi-Square = 252.36 and Degrees of Freedom = 156 which gave a χ2/df = 1.61. For non-centrality fit indices, the Steiger-Lind RMSEA was 0.09 [0.025–0.235]. Moreover, the Joreskog GFI equaled 0.903 and AGFI equaled 0.916.

#### Bivariate analysis

Adolescents whose parents are separated showed a significantly higher score on victimization of bullying in comparison to those whose parents live together (9.52 vs. 6.19). Furthermore, higher social phobia (*r* = 0.320) and avoidance (*r* = 0.173), internet addiction (*r* = 0.342), psychological (*r* = 0.500), neglect (*r* = 0.318), physical (*r* = 0.439) and sexual (*r* = 0.400) abuse were significantly associated with higher victimization of bullying score, whereas higher physical activity (*r*=-0.076) was significantly associated with lower victimization of bullying score (Table 3).

**Table 3:** Bivariate analysis taking the victimization of bullying scale as the dependent variable in the whole sample

	Victimization of bullying scale	*P* –value
	**Mean ± SD**	
**Parents status**		
Living together	6.19 ± 7.16	**< 0.001**
Separated	9.52 ± 6.13	
	**Correlation coefficient*r***	***p***
Liebowitz- fear score	0.320	**< 0.001**
Liebowitz- avoidance score	0.173	**< 0.001**
Internet addiction	0.342	**< 0.001**
House crowding index	-0.019	0.447
Physical activity score	-0.076	**0.003**
Depression score	0.179	**< 0.001**
Psychological abuse scale	0.500	**< 0.001**
Child abuse neglect scale	0.318	**< 0.001**
Child abuse physical scale	0.439	**< 0.001**
Child abuse sexual scale	0.400	**< 0.001**

Numbers in bold indicate significant *p*-values

#### Multivariable analysis

The results of a first stepwise linear regression, taking the bullying victimization score as the dependent variable, showed that higher psychological abuse (Beta = 0.16), child physical (Beta = 0.19) and neglect (Beta = 0.08), higher internet addiction (Beta = 0.10) and higher social fear (Beta = 0.04) were significantly associated with higher bullying victimization score. Higher physical activity was significantly associated with lower bullying victimization score (Beta=-0.05) (Table 4, Model 1).

**Table 4:** Multivariable analysis

**Model 1: Linear regression taking the bullying victimization score as the dependent variable**					
	**Unstandardized Beta**	**Standardized Beta**	p	**95% CI**	
				**Lower**	**Upper**
Psychological abuse	0.165	0.258	< 0.001	0.125	0.206
Internet addiction	0.105	0.270	< 0.001	0.088	0.122
Child abuse physical	0.197	0.176	< 0.001	0.131	0.263
Child abuse neglect	0.083	0.117	< 0.001	0.052	0.113
Physical activity index	-0.052	-0.107	< 0.001	-0.073	-0.032
Liebowitz- fear	0.045	0.105	< 0.001	0.024	0.066
**Model 2: Logistic regression taking being bullied vs. not being bullied as the dependent variable**					
		**ORa**	**p**	**95% CI**	
				**Lower**	**Upper**
Parents status (separate vs. living together^a^)		3.084	< 0.001	1.958	4.858
Mild internet addiction vs. normal^a^ use		4.719	< 0.001	3.304	6.738
Moderate internet addiction vs. normal^a^ use		3.840	< 0.001	2.633	5.600
Poor physical activity vs. sedentary^a^		0.200	< 0.001	0.131	0.307
Fair physical activity vs. sedentary^a^		0.940	0.858	0.479	1.847
Very good physical activity vs. sedentary^a^		0.043	< 0.001	0.013	0.142
Liebowitz- fear (high vs. low^a^)		1.507	0.024	1.055	2.152
Liebowitz- avoidance (high vs. low^a^)		0.496	< 0.001	0.344	0.715
Psychological abuse scale (high vs. low^a^)		3.596	< 0.001	2.633	4.910
Child abuse neglect scale (high vs. low^a^)		2.217	< 0.001	1.658	2.965
Child abuse physical scale (high vs. low^a^)		4.554	< 0.001	3.310	6.264

For Model 1: Variables entered in the model: parents’ status, IAT score, Liebowitz fear score, Liebowitz avoidance score, psychological abuse scale, child abuse neglect scale, child abuse physical scale and child abuse sexual scale, depression and physical activity index.

Adjusted *R*^2^ = 0.383.

For Model 2: Variable(s) entered on step 1: Parents status, smoking status, Internet addiction test, Liebowitz fear score, Liebowitz avoidance score, psychological abuse scale, child abuse neglect scale, child abuse physical scale, child abuse sexual scale, house crowding index and physical activity score.

Adjusted *R*^2^ = 0.517.

^a^Reference group

The results of a second logistic regression, taking being bullied vs. not being bullied as the dependent variable, showed that having a separate parents (ORa = 3.08), Mild (ORa-4.71) to moderate (ORa = 3.84) internet addiction test, higher social fear (ORa = 1.50), higher psychological abuse (ORa = 3.59), higher child neglect (ORa = 2.21) and physical (ORa = 4.55) abuse were significantly associated with higher odds of being bullied. However, higher social avoidance (ORa = 0.49), poor (ORa = 0.20), fair (ORa = 0.94) and very good (ORa = 0.04) physical activity as compared to sedentary were significantly associated with lower odds of being bullied (Table 4, model 2).

## Discussion

This is the first study conducted in Lebanon investigating the association between child abuse, Internet addiction, social phobia, depression and bullying victimization among Lebanese adolescents, in addition to the previously associated factors with bullying victimization in Lebanon [29, 30]. Our study found that around 49.9% of adolescents were victims of bullying. The current study showed that child abuse (psychological, physical, sexual and neglect), Internet addiction, social phobia and avoidance were positively correlated with bullying victimization.

The results of the linear regression were confirmed by the logistic regression and the following variables psychological abuse, child physical and neglect abuse, internet addiction, and social fear were encountered in both models and showed higher effect on bullying victimization. However, physical activity was common in both regressions and found to be associated with lower risk of bullying victimization. The only difference between the two regression models was that separated parents and higher social avoidance were encountered only in logistic regression and were associated with higher and lower effect on bullying victimization respectively.

The findings of our study showing a lower risk of bullying development if physical activity was practiced is consistent with the findings of other studies [70, 71]. It has been reported that physical activity is a vital means for the transmission of values, aids in communication skills, and promotes prosocial attitudes [72]. Performance of the physical activity has been suggested to be a health-enhancing behavioral practice and it has been perceived that physically active student have the capacity to protect themselves [73]. Thus, physical activity can be helpful tools in the prevention and treatment of bullying and have a lower risk of developing aggressive and deviant behaviors.

Our study showed that separated parents increased the risk of being bullied, which is consistent with the results of other studies [24, 74, 75]. Children whose parents are separated are exposed for the lengthy lack of care and nurturance from their parents that makes them more vulnerable to bullying behavior, resulting in frequent bullying [76]. It is plausible that children who do not have parents in their daily lives, they lack parental care and companionship, security, or self-confidence, and are thus more likely to become subject to bullying [76].

### Illinois Bully Scale validation in Lebanon

In order to examine bullying victimization among Lebanese adolescents, the Illinois Bully Scale was validated in our study. The demonstrated Cronbach’s *α* showed high internal consistency and reliability (α_Cronbach_ *=* 0.955) in contrast with the initial scale’s reliability (α_Cronbach_ = 0.87) [60], the validated version for the Pakistani population (α_Cronbach_ = 0.88) [77], as well as the Iranian version (Cronbach’s α = 0.81) [46]. We obtained two factors through the scale’s items while the validated version in Pakistan [77], while the original and Persian scales’ items converged into three factors [46, 60].

### Prevalence of bullying victimization in Lebanon

49.9% of adolescents in our study were victims of bullying, consistent with the previous data that documented prevalence of around 45–59% in Lebanon [52]. The high rates of bullying victimization in Lebanon can be correlated with the lack of anti-bullying rules at schools, teachers not taking immediate action to stop bullying among peers in classes, absence of bullying prevention programs and awareness among schools’ academic and administrative staff about its impact.

### Effect of social phobia and avoidance on bullying victimization

Our study shows that social phobia and avoidance are highly linked with a greater bullying victimization risk, consistent with the results of previous studies [78, 79]. The underlying explanation for the relation between social phobia and bullying victimization is that extreme fear of being present in social settings, interpersonal communication and being assessed by people develop negative beliefs about oneself [80]. Social phobia results in public evasion in affected individuals, reducing peer communication, therefore affecting peer communication, prolonging bullying victimization and decreasing self-confidence; these consequences are caused by a belief of being avoided by others [81]. In addition, adolescents addicted to Internet use suffer from social impairments causing community loneliness, difficulty interacting with family members, insecurity, and low self-esteem, which subsequently increase the vulnerability for bullying victimization [82]. Our results support the fact that adolescents who were exposed to abuse are at higher risk of being bullied; several authors documented that emotional dysregulation that develops in response to abuse, whether at home or between peers, impairs social interactions and results in internalizing problems such as anxiety and depression, and externalizing behaviors that could include conduct disorder and substance use [5, 83]. In conclusion, avoidance and isolation interfere with effective social integration of adolescents, making them highly prone to being victimized by others [84].

### Effect of Internet addiction on bullying victimization

The association found in our results between Internet addiction and bullying victimization is in agreement with previous findings [23]. The literature found that emotional difficulties, misconduct, hyperactivity, peer problem behaviors and social isolation are mediating factors between Internet addiction and bullying victimization [23]. Another study revealed that Internet addiction, depression and substance abuse were highly linked with bullying victimization [85]. Additionally, Internet addiction predicted both traditional and cyberbullying victimization [86]. Increased Internet use is associated with many adverse consequences, which include Internet addiction, bullying perpetration and increased chances of bullying victimization [87].

### Effect of child abuse on bullying victimization

Child sexual abuse was significantly associated with higher bullying victimization, which is consistent with previous results [32]. In Lebanon, it was found in a previous study that around 48% reported at least one incident of both physical and psychological abuse, which shows that abuse prevalence is high among Lebanese children [88]. The direct and positive association between child sexual abuse and bullying victimization can be explained by the fact that adolescents who are sexually abused are more likely to experience subsequent sexual, psychological, and physical victimization [89, 90]. In addition, studies have demonstrated that a feeling of betrayal, shame and stigmatization usually follows child sexual abuse, which causes a substantial impact on interpersonal functioning and increases the vulnerability to bullying victimization [91, 92]. Child neglect is associated with a higher risk of bullying victimization, consistent with previous literature. Emotional neglect creates parental attachment problems and communication difficulties with peers [93]. Similarly, physical child abuse increases the risk of bullying victimization. Physical child abuse exerts detrimental effects on the relationship between adolescents and parents, and distorts the victim’s perception of stressful situations [33]. People who have been exposed to physical abuse also experience sentiments of disgrace and suffer from interpersonal difficulties including being bullied by others [33, 94].

### Effect of house crowding index on bullying

In this study, it was shown that a high household crowding index was associated with lower risk of bullying victimization. Our results are in conflict with previous research; high house crowding index was previously shown to affect adolescents’ wellbeing by causing discomfort, an inability to perform daily activities, an unstable sleep pattern, a difficulty in concentrating, a low mood and negative behavior, which may cause a higher bullying victimization vulnerability [95]. Another study found that both younger and older individuals living in crowded residential areas and households were more likely to be socially isolated and bullied by others [96]. The negative correlation between bullying and house crowding index can be explained by the fact that bullying victimization differs according to level of education, despite crowded living situations; both bullying victimization and perpetration correlated negatively with level of education in a crowded area [97]. This inconsistency with literature proposes further investigation on the relation between bullying victimization and household crowding index. Having said that, house crowding can be indicative of low socioeconomic status, which is connected with bullying victimization among adolescents [98]. A meta-analysis including multiple studies also showed that victims of bullying were more likely to come from low socioeconomic households [99].

### Clinical implications

It is important to investigate the presence of different factors that might be associated with bullying victimization. Adolescents suffering from bullying victimization should be supported using a behavioral therapy approach, in order to enhance critical insight, learn techniques for adequate communication with peers and prevent the possible detrimental consequences. Victims of bullying must be introduced to healthier coping strategies to decrease fear and avoidant behavior. Additionally, school staff members, especially teachers, have a fundamental role in preventing and intervening during bullying incidents in educational settings. The adoption and application of bullying preventive programs in Lebanese schools are needed to optimize interactions, minimize bullying, and reduce aggressive behavior in schools among adolescent students [100].

### Limitations

Our study has some limitations, such as the possibility of biased answers due to the self-reported measures. The study used a cross-sectional design, which hinders it from determining causality. The relationship between bullying victimization and other factors such as child sexual abuse may be bidirectional [101], proposing further studies with a longitudinal design. In addition, the self-reported experience of bullying victimization and other associated factors may result in recall bias. Our findings from school students may not be generalized to adolescents that have dropped out from school. Furthermore, we are aware of the probability that some questions might not have been completely understood by the participants, along with the possibility that not all students took the questionnaire in a serious manner. We might also consider selection bias during the selection of schools in certain regions and because public schools were not enrolled. It is important to mention the social desirability bias; respondents might have answered questions in a way that portrays them favorably. Finally, some sociodemographic characteristics were not collected such as ethnicity, sexuality, presence of a disability, and family wealth as a particularly important social factor.

Considering the limitations of the study, more research is required, which takes the missing variables into account.

## Conclusions

Our findings attest that bullying victimization is likely to be associated with certain factors such as child abuse of all forms, Internet addiction and social phobia and avoidance. Also, the Illinois Bully Scale was validated in Lebanon for the first time. More attention should be paid to students vulnerable to bullying victimization, such as those with environmental or domestic problems, and adolescents with psychological disorders such as behavioral addictions. Since the present study is the first step in understanding the relationship between the factors associated with bullying victimization among Lebanese adolescents, future studies are needed to further investigate factors that may predict bullying victimization.

## Data Availability

All data generated or analyzed during this study is not publicly available to maintain the privacy of the individuals’ identities. The dataset supporting the conclusions is available upon request to the corresponding author.

## Abbreviations

LSAS: Liebowitz Social Anxiety Scale
IAT: Internet Addiction Test
ADRS: Adolescent depression rating scale
CAS: Child abuse self-report scale
KMO: Kaiser-Meyer-Olkin
RMSEA: Root Mean Square Error of Approximation
GFI: Goodness of Fit Index
AGFI: Adjusted Goodness of Fit Index
